# Cortical compensation mechanism for swallowing recovery in patients with medullary infarction-induced dysphagia

**DOI:** 10.3389/fneur.2024.1346522

**Published:** 2024-07-31

**Authors:** Furong Gu, Jing Han, Qiang Zhang, Xiangyu Li, Yue Wang, Jialing Wu

**Affiliations:** ^1^Clinical College of Neurology, Neurosurgery and Neurorehabilitation, Tianjin Medical University, Tianjin, China; ^2^Department of Neurology, Tianjin Huanhu Hospital, Tianjin Key Laboratory of Cerebral Vascular and Neurodegenerative Diseases, Tianjin Neurosurgical Institute, Tianjin, China; ^3^Department of Neuroradiology, Tianjin Huanhu Hospital, Tianjin, China; ^4^Department of Otolaryngology, Tianjin Huanhu Hospital, Tianjin, China; ^5^Department of Rehabilitation, Tianjin Huanhu Hospital, Tianjin, China

**Keywords:** dysphagia, medullary infarction, fMRI, swallowing rehabilitation, FEES, saliva swallowing

## Abstract

**Introduction:**

This study aims to examine brain activity during different swallowing actions in patients with dysphagia caused by medullary infarction (MI) before and after treatment using blood oxygen level-dependent (BOLD) functional magnetic resonance imaging.

**Methods:**

Fifteen patients were enrolled in this study. Brain activation during saliva swallowing and effortful saliva swallowing was observed using BOLD imaging in the acute phase of stroke and after 4 weeks of rehabilitation training. Differences in the activation of brain regions during saliva swallowing before and after treatment, during effortful saliva swallowing before and after treatment, and between the two swallowing actions before and after treatment were compared.

**Results:**

In the acute phase of stroke, only the bilateral precentral and left lingual gyrus were partially activated during saliva swallowing, and there was no obvious activation in the insula. Effortful saliva swallowing activated more brain regions than saliva swallowing before treatment, including the bilateral supplementary motor area (SMA), postcentral gyrus, and right insular cortex. The number of brain regions activated during saliva swallowing increased after treatment, including the bilateral precentral gyrus, postcentral gyrus, insula, thalamus, and SMA.

**Discussion:**

Cortical activation increases after recovery from dysphagia, and the increased activation of the postcentral gyrus might play a functional compensatory role. Effortful saliva swallowing is a more effective rehabilitation training method for patients with dysphagia caused by MI.

## 1 Introduction

Dysphagia is a common manifestation in stroke patients, occurring in ~25–45% of acute stroke cases, and severe dysphagia is more likely to occur in patients with medullary infarction (MI) (1). Dysphagia can result in malnutrition, increase the risk of aspiration pneumonia, hinder the recovery of patients with stroke, prolong hospitalization, and increase the incidence of adverse outcomes and mortality. Early detection and treatment of dysphagia can reduce subsequent complications, shorten hospital stays, and improve prognosis.

Blood oxygen level-dependent (BOLD) functional magnetic resonance imaging (fMRI) is based on the difference in paramagnetism between oxygenated and deoxygenated hemoglobin and the principle of increased oxygen demand during neuronal activity. This technique reflects the changes in brain activity in various functional brain regions. Owing to the efforts of many experts, BOLD technology has developed rapidly in the past 30 years (2). BOLD-fMRI studies have demonstrated that the cortical regions related to swallowing in healthy individuals include the precentral gyrus, postcentral gyrus, supplementary motor area (SMA), anterior cingulate cortex, insula, frontal gyrus, temporal gyrus, and cuneus, and the activated brain regions differ depending on the specific swallowing actions or types of food (3–6).

In patients with dysphagia after stroke, the brain regions that are activated during swallowing movements change. Because fMRI scanning requires the swallowing action to be performed in the supine position, thereby increasing the risk of choking during swallowing in patients with dysphagia, there are relatively few BOLD imaging studies on patients with dysphagia. Dysphagia can be caused by damage to the brainstem, cortex, and subcortical structures, which result in similar clinical manifestations. At present, current research findings on the activated regions and compensatory mechanisms during swallowing after stroke lack consistency. This may be because the studies failed to differentiate the damaged site or type of swallowing disorder. We aimed to select patients with dorsolateral medullary infarction whose clinical manifestations and Fiberoptic Endoscopic Evaluation of Swallowing (FEES) results were consistent with those of pharyngeal phase dysphagia to minimize variability between participants in the group and obtain more consistent conclusions.

Swallowing rehabilitation is a pivotal intervention for treating dysphagia. However, the cortical compensatory mechanism of rehabilitation training for improving swallowing function and which rehabilitation method is better for specific patients remain uncertain. Therefore, this study aimed to investigate the cortical activation during saliva swallowing and effortful saliva swallowing using BOLD imaging in patients with dysphagia shortly after MI and after dysphagia recovery to explore the cortical compensatory mechanism of dysphagia recovery.

## 2 Materials and methods

### 2.1 Participants

We recruited patients with acute stroke of the dorsolateral medulla who met the following criteria and were hospitalized at the Department of Neurology of Tianjin Huanhu Hospital between January 2022 and June 2022. The inclusion criteria were as follows: (i) first-time stroke with confirmed lesions involving the dorsolateral medulla on MRI within 48–72 h of onset; (ii) age between 30 and 75 years; (iii) no history of swallowing disorders before stroke but presenting with dysphagia after stroke, with a score of 3–4 on the Kubota Water Swallowing Test; (iv) clear consciousness, a Mini-Mental State Examination (MMSE) score >24, stable vital signs, and ability to cooperate with the functional examination and swallowing rehabilitation training confirmed by simulated measurements; and (v) voluntarily participated in the study and provided written informed consent.

The exclusion criteria were as follows: (i) dysphagia caused by other neurological diseases or organic lesions; (ii) concomitant cerebral infarction in other brain regions, including the cortex, thalamus, and cerebellum; (iii) patients with metal or other implants who could not undergo 3.0 T MRI; (iv) patients with lesions in the oral cavity, nasal cavity, pharynx, and larynx who could not undergo FEES; and (v) patients with obvious hiccups or severe dysphagia who could not complete the supine swallowing action and those with unstable conditions who were not able to undergo fMRI.

Fifteen patients with an age range of 35–75 years and average age of 54 years were included in the study, and three patients were female. Based on the Edinburgh Handedness Inventory (7), all 15 patients were all right handed before stroke onset (Table 1).

**Table 1:** Patient characteristics.

**Patient**	**Age**	**Gender**	**Time after (stroke hours)**	**Lesion side**	**Score of water swallowing test at enrollment**	**Score of water swallowing test after treatment**
1	75	M	50	L	4	2
2	37	M	52	L	3	1
3	35	M	53	L	3	1
4	65	F	52	R	4	1
5	48	F	53	L	4	2
6	70	M	56	R	3	1
7	48	M	60	R	4	1
8	51	M	57	R	4	2
9	54	M	53	L	3	1
10	55	F	56	R	3	1
11	60	M	60	R	4	2
12	52	M	65	L	3	1
13	59	M	66	R	3	1
14	50	M	60	L	3	1
15	51	M	67	R	3	1

Patient characteristics including age, gender, time after stroke at the first fMRI study (hours), lesion side, and score of Kubota Water Swallowing Test.

This study was conducted in accordance with the Declaration of Helsinki. The studies involving human participants were reviewed and approved by the Ethics Committee of Tianjin Huanhu Hospital, with approval number (Jinhuan) No. (2020-55). The patients provided their written informed consent to participate in this study.

### 2.2 Swallowing function evaluation, FEES, and rehabilitation training methods

FEES and BOLD scanning were performed after enrollment, and swallowing rehabilitation training was provided 5 days a week. Swallowing function evaluation, FEES, and BOLD scanning were performed again after 4 weeks of treatment.

#### 2.2.1 Swallowing function evaluation

Swallowing function was assessed using the Kubota Water Swallowing Test (8), wherein the participants drank 30 mL of warm water while seated. The classification criteria were as follows: Grade 1, smooth swallowing of water once; Grade 2, swallowed in two parts without choking; Grade 3, swallowed at once but experienced choking; Grade 4, swallowed in more than two parts with choking; and Grade 5, frequent coughing and inability to swallow.

#### 2.2.2 FEES method

The patients were seated, and topical anesthesia (1% lidocaine + 1/200,000 adrenaline solution) was administered. A fiberoptic endoscope was passed along the floor of the nose through the velopharyngeal port into the pharynx, and the anatomy of the nasopharynx, tongue base, hypopharynx, larynx, and vocal cords, as well as the accumulation of secretions, were observed. We tested the sensation in the throat when the probe touched the arytenoid cartilage. We asked the patient to phonate a high-pitched “eee” to observe the contraction of the pharyngeal muscle and elevation of the larynx. The endoscope was then passed into the hypopharynx, and the patients were asked to swallow fluid (orange juice), semi-solid food (plain yogurt), and solid food (soft bread) in turn to assess leakage, penetration, retention, aspiration, and choking.

#### 2.2.3 Swallowing rehabilitation training

In addition to conventional stroke treatment, various swallowing rehabilitation training methods, including biological low-frequency electrical stimulation of swallowing, cold stimulation training, saliva swallowing training (dry swallow, saliva swallowing action during BOLD scanning) (8), Masako training (effortful saliva swallowing during BOLD scanning, wherein participants squeeze the muscles of the back of their tongue and swallow his saliva as hard as they can) (8), Shaker exercises, and the Mendelsohn maneuver. These methods were utilized to increase sensory stimulation of the pharynx and tongue root strength, improve the contractile force of the pharyngeal muscle, increase the opening time and width of the cricopharyngeal muscle, increase the opening of the upper esophageal sphincter, and improve the sensory and motor function during swallowing (8). Considering the need for a supine examination, two training methods—saliva swallowing and effortful saliva swallowing—were selected for BOLD scanning.

### 2.3 Magnetic resonance imaging

A 3.0 T MR scanner (Magnetom Skyra, Siemens, Germany) with a 20-channel head coil was used to obtain functional and structural images. Patients were positioned supine on the scanner bed, with their heads fixed in a birdcage-shaped coil, and they viewed the experimental tasks on a screen through a mirror mounted on the head coil. Foam earplugs and pads were used to reduce scanner noise and head motion, respectively. High-resolution T1-weighted structural imaging, functional imaging, and diffusion-weighted imaging (DWI) were performed sequentially, with the scanning plane parallel to the anterior-posterior commissure line. The structural (T1) image parameters were as follows: TR, 2,000 ms; TE, 2.98 ms; matrix, 256 × 256; field of view, 256 × 256 mm; slice thickness, 1 mm; and 192 slices. DWI was performed using the following parameters: TR, 5,200 ms; TE, 80 ms; field of view, 240 mm × 240 mm; 36 slices; and slice thickness, 4 mm. Functional imaging was performed using the magnetic-sensitive GRE-EPI BOLD contrast T2^*^WI imaging sequence, with the following parameters: TR, 2,000 ms; TE, 30 ms; excitation time, 1; flip angle 90°; field of view, 220 mm × 220 mm; matrix, 64 × 64; 36 slices; slice thickness, 4 mm; no slice gap; and imaging time, 370 s; a total of 90 dynamics were acquired to cover the entire frontal lobe cortex to the level of the medulla oblongata.

### 2.4 BOLD paradigm

**Figure 1:**
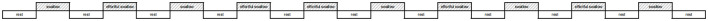
Specific task sequence of swallowing during BOLD scanning, each block lasts 18s.

BOLD paradigm is shown in Figure 1. An initial blank resting module of 18 s was set to eliminate the influence of blood flow signal saturation and the magnetic environment. A final blank module of 10 s was set to avoid the final effect of nuclear magnetism. The task employed a BLOCK experimental design, with saliva swallowing and effortful saliva swallowing presented in a pseudorandom manner through visual text prompts. The participants were instructed to perform as many swallowing motions as possible during the swallowing cue period. During the black screen period, the participants rested quietly without any other movements except for breathing. Figure 1 presents the specific task sequence, with each set of swallowing actions lasting for 18 s, followed by an 18 s rest period for a total of 10 sets. The final 10 s blank module was also included, resulting in a total time of 370 s. During BOLD scanning, the experimenters accompanied the participants to the MRI room and observed their swallowing actions to ensure that they followed the visual cue requirements for swallowing and resting.

### 2.5 BOLD image processing

All fMRI data were preprocessed using SPM 12 (Statistical Parametric Mapping, Wellcome Department of Imaging Neuroscience, London, UK) running on MATLAB (MathWorks, Natick, MA). The first 10 time points were removed to eliminate the possibility of unstable AMRI signals. If a patient's motion and rotation parameters exceeded 1.5 mm and 1.5°, respectively, this run of data was excluded from future analysis. The images were subsequently spatially normalized to the Montreal Neurological Institute template brain, resampling voxel size = 3 × 3 × 3 mm^3^. Functional images were spatially smoothed with a three-dimensional Gaussian kernel of 6 mm full width at half maximum to increase the signal-to-noise ratio and reduce inter-subject differences.

### 2.6 Statistical analysis

The experimental data were organized into a database using Excel, and the SPSS statistical software package version 20.0 (SPSS Inc., Chicago, IL, USA) was used to analyze the activation volume and intensity of the brain functional areas. The General Linear Model (GLM) was used in the first-level analysis.The activated brain regions and their voxels in each participant were tested using one sample *t*-test, To observe the activation in each action, we defined saliva swallowing vs. rest period as con1, effortful saliva swallowing vs. rest period as con2, effortful saliva swallowing minus saliva swallowing as con3. In the second-level analysis, the statistical analysis, the beta weights of two contrasts were statistically compared separately using one sample *t*-test, with the age and sex of participants as covariate. The results were corrected for multiple comparisons using the false discovery rate (FDR) correction at the voxel-level, and the significance threshold was set at FDR-corrected *P* < 0.01 with a minimum cluster size (the number of voxels) of 10 voxels.

## 3 Results

### 3.1 General information and FEES results

**Figure 2:**
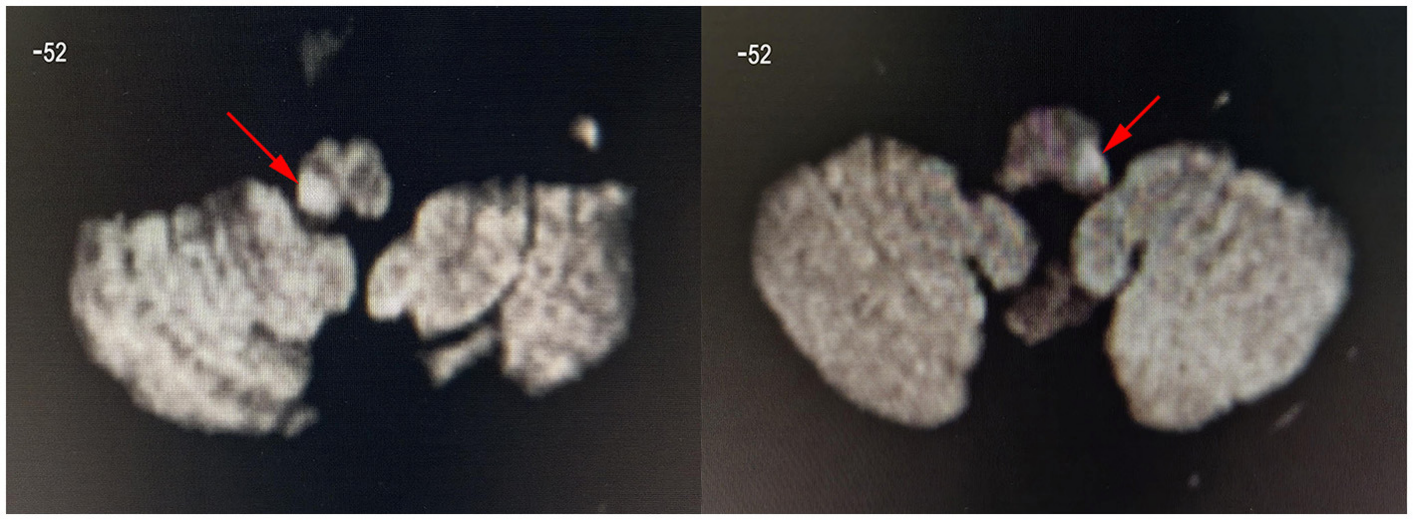
Diffusion-weighted images showing dorsolateral medulla infarction (arrow). These are the DWI images of patient 8 (left image, lesion located in the right medulla oblongata) and patient 1 (right image, lesion located in the left medulla oblongata). The z-value of the coordinate for the slice position is −52. All lesions were located in the left or right side of dorsolateral medulla oblongata.

Table 1 provides an overview of the clinical and demographical characteristics of our patients. All lesions were located on the left or right side of the dorsolateral medulla and were confirmed using MRI (Figure 2). After enrollment, DWI scans were conducted along with two BOLD scans before and after rehabilitation, and no new lesions were observed in comparison to the DWI images prior to inclusion in the study. The score of Kubota Water Swallowing Test after rehabilitation training (1.267 ± 0.4577) was significant lower than that at enrollment (3.400 ± 0.5071; *P* < 0.001, Table 1).

FEES was performed after enrollment (2–3 days after the onset of stroke), revealing impaired pharyngeal swallowing function, indicated by vocal cord paralysis, valleculae and pyriform fossa retention of a saliva-like substance, absence of swallowing reflex upon touching the epiglottis cartilage and posterior pharyngeal wall with a touch stick, a delayed swallowing response, residual vallecular material observed when swallowing viscous food and bread, and leakage and aspiration in some patients. The residual material was removed after repeated swallowing.

After rehabilitation training for 4 weeks, five patients declined follow-up FEES due to their perception of swallowing without any difficulty, and 10 patients underwent a follow-up FEES. Examination of the 10 patients revealed significantly improved pharyngeal swallowing function; there was no vocal cord paralysis and no obvious retention of saliva-like substances in the valleculae or pyriform fossa. In some patients, the swallowing reflex was still delayed upon touching the epiglottis cartilage and posterior pharyngeal wall with a touch stick. Notably, leakage or aspiration was not observed.

### 3.2 BOLD results for different swallowing actions before and after treatment

#### 3.2.1 Brain regions activated during saliva swallowing in the acute stroke period

**Figure 3:**
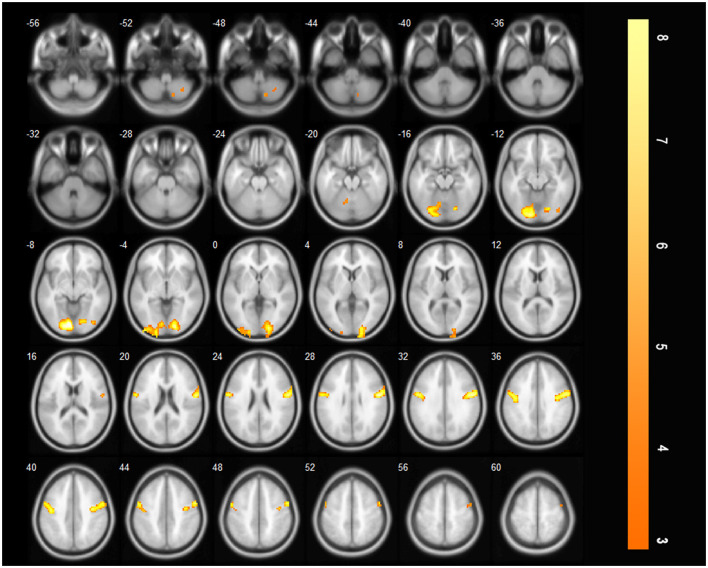
Only the left lingual gyrus and bilateral precentral gyrus were activated during saliva swallowing in the acute stroke period (*P* < 0.01, FDR corrected, cluster size > 10 voxels).

After acute stroke, less activation of the brain regions was observed during saliva swallowing, and only the left lingual gyrus and bilateral precentral gyrus were activated. Low-intensity activation was observed in the right inferior occipital lobule, right inferior semilunar lobule, and right posterior lobe of the cerebellum, as shown in Figure 3 and Table 2.

**Table 2:** Brain regions activated during saliva swallowing in the acute stroke period.

**Regions (AAL)**	**Cluster size**	**Peak *T*-value**	**MNI coordinate**		
			**X**	**Y**	**Z**
Lingual _L	321	7.8231	−16	−54	4
Precentral_R	267	7.2283	39	3	45
Precentral_L	208	6.3082	−36	6	45
Occipital Inf_R	19	4.5953	24	−92	−6
Cerebellum 3_R	14	3.5837	8	−42	−18

Cluster size, the number of voxels; MNI, Montreal Neurological Institute; R, right; L, left; P < 0.01, FDR corrected, cluster size > 10 voxels.

#### 3.2.2 Brain regions activated during effortful saliva swallowing in the acute stroke period

In the acute stroke period, more brain regions were activated during effortful saliva swallowing, including the left lingual gyrus, bilateral precentral gyrus, postcentral gyrus, and SMA, along with low-intensity activation in the right insula, bilateral posterior cerebellum, right inferior temporal gyrus, as shown in Table 3.

**Table 3:** Brain regions activated during effortful saliva swallowing in the acute stroke period.

**Regions(AAL)**	**Cluster size**	**Peak T-value**	**MNI coordinate**		
			**X**	**Y**	**Z**
Cerebellum 3_R	40	5.1808	8	−42	−18
Cerebellum 3_L	39	4.7408	−8	−42	−18
Fusiform_R	56	5.5977	30	−38	−10
Lingual_L	480	7.5498	−16	−54	4
Occipital_Inf_R	16	4.1862	24	−92	−6
Precentral_R	445	9.1773	39	3	45
Postcentral_R	190	4.3643	54	−21	36
Insula_R	12	4.0366	34	16	10
Precentral_L	330	8.4933	−36	6	45
Postcentral_L	219	6.4920	−54	−18	36
Supp_Motor_Area_L	145	6.2439	−6	12	60
Supp_Motor_Area_R	13	4.4967	6	12	60

Cluster size, the number of voxels; MNI, Montreal Neurological Institute; R, right; L, left; P < 0.01, FDR corrected, cluster size > 10 voxels.

#### 3.2.3 More brain regions were activated during effortful saliva swallowing than during saliva swallowing in the acute stroke period

**Figure 4:**
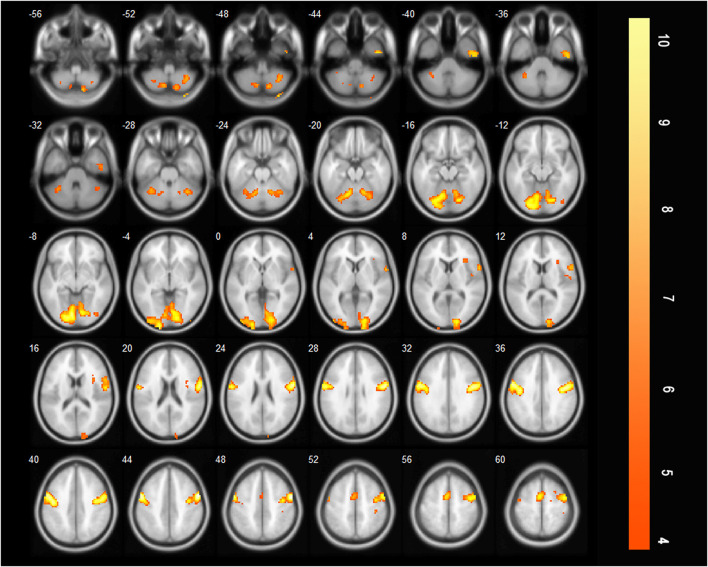
T-score map showing difference between two swallowing actions (effortful saliva swallowing minus saliva swallowing) in the acute stage of stroke. Activation of the bilateral SMA, postcentral gyrus, right insula, and the bilateral precentral gyrus can be seen (*P* < 0.01, FDR corrected, cluster size > 10 voxels).

The additionally activated regions during effortful saliva swallowing (effortful saliva swallowing minus saliva swallowing) included the bilateral SMA, postcentral gyrus, and right insula, with increased voxel numbers in the bilateral precentral gyrus compared with those regions activated with saliva swallowing in the acute stroke period (Figure 4).

#### 3.2.4 Brain regions activated during saliva swallowing after treatment

After treatment, more brain regions were activated during saliva swallowing, including the left lingual gyrus, bilateral precentral and postcentral gyrus, SMA, insula, thalamus, and low-intensity activation in left inferior frontal gyrus, left calcarine, which are similar to the brain regions activated during saliva swallowing in healthy individuals, as shown in Table 4.

**Table 4:** Brain regions activated during saliva swallowing after treatment.

**Regions(AAL)**	**Cluster size**	**Peak T-value**	**MNI coordinate**		
			***X***	***Y***	***Z***
Lingual_L	340	7.4724	−16	−54	4
Occipital_Inf_R	19	4.5953	24	−92	−6
Cerebellum 3_R	14	3.5837	8	−42	−18
Frontal_inf_R	35	3.7695	26	36	−16
Calcarine_L	42	5.1877	−10	−92	4
Insula_R	53	5.3279	34	16	10
Thalamus_L	15	4.2452	−14	−18	8
Parahippocampal_L	22	4.0066	−24	−8	−32
Insula_L	27	5.1806	−34	10	10
Precentral_R	579	11.0163	39	3	45
Postcentral_R	182	4.8112	56	−21	36
Precentral_L	479	12.7845	−36	6	45
Postcentral_L	413	6.7225	−63	−9	30
Thalamus_R	21	5.1329	18	−18	8
Supp_Motor_Area_L	72	3.5691	−6	12	72
Supp_Motor_Area_R	76	5.0964	9	12	72

Cluster size, the number of voxels; MNI, Montreal Neurological Institute; R, right; L, left; P < 0.01, FDR corrected, cluster size > 10 voxels.

#### 3.2.5 Brain regions activated during effortful saliva swallowing after treatment

**Figure 5:**
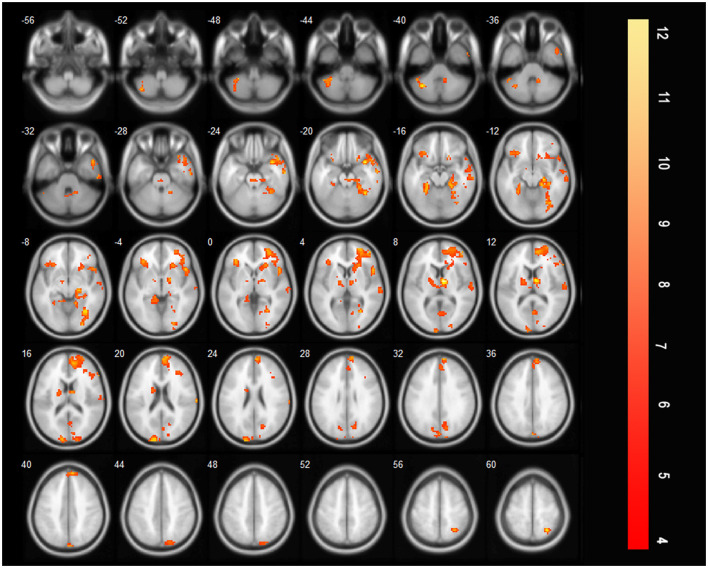
T-score map showing many brain areas activated during effortful saliva swallowing after treatment in axial slices, including bilateral precentral and postcentral gyrus, SMA, insula, thalamus, and bilateral supramarginal gyrus (*P* < 0.01, FDR corrected, cluster size > 10 voxels).

After treatment, there are many brain areas activated during effortful saliva swallowing, including the right cerebellum, right superior temporal gyrus, bilateral precentral and postcentral gyri, SMA, insula, thalamus, and bilateral supramarginal gyrus. These regions were similar to those activated in healthy individuals during effortful saliva swallowing (Figure 5).

#### 3.2.6 More brain regions were activated during saliva swallowing after treatment compared with the acute stroke period

**Figure 6:**
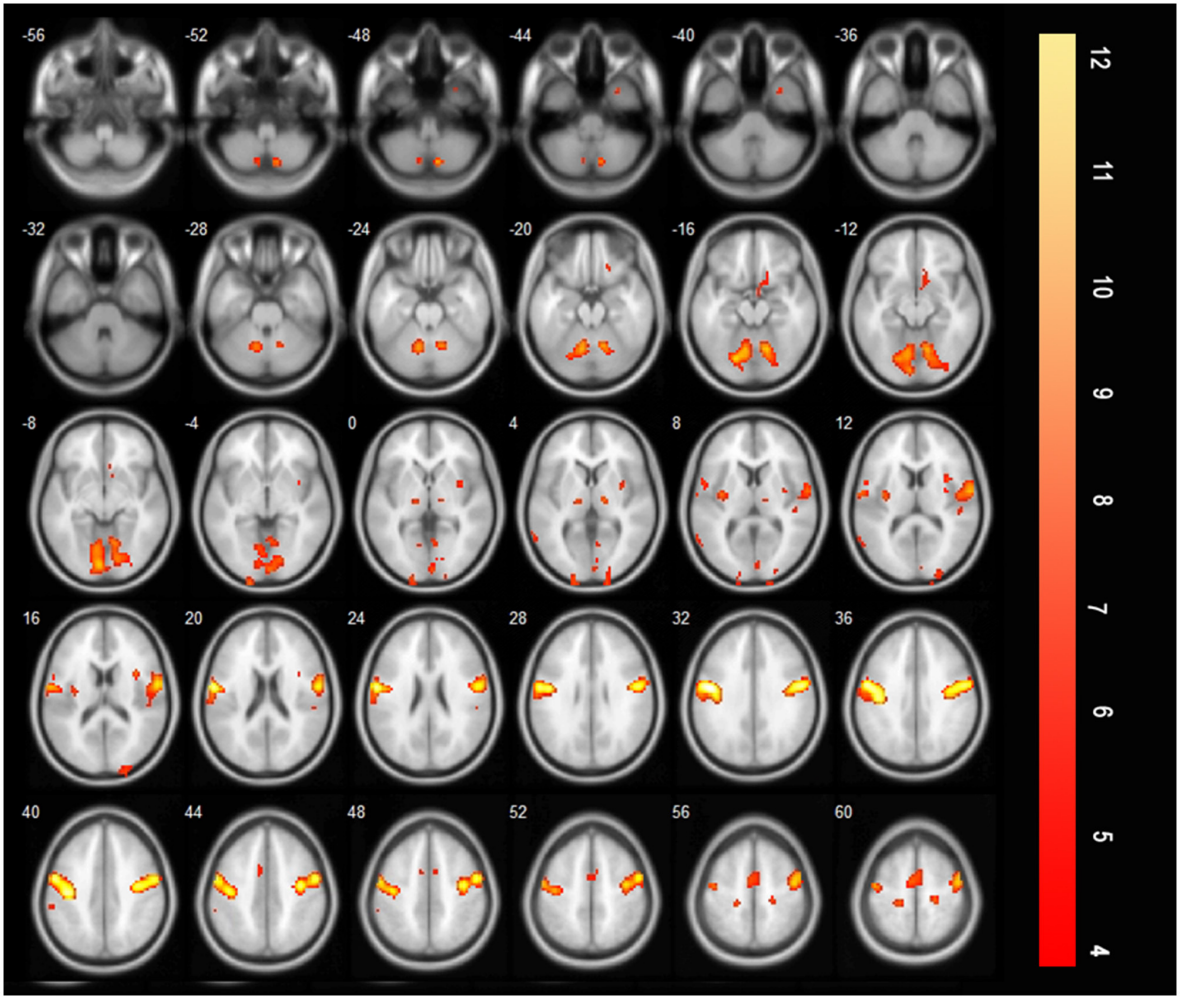
T-score map showing the difference during saliva swallowing between two timepoints (saliva swallowing after treatment minus saliva swallowing in the acute stroke period), the bilateral precentral and postcentral gyrus, bilateral SMA, bilateral insula, and bilateral thalamus were additionally activated (*P* < 0.01, FDR corrected, cluster size > 10 voxels).

Compared with those before treatment, the bilateral precentral and postcentral gyri, bilateral SMA, bilateral insula, and bilateral thalamus were additionally activated during saliva swallowing after rehabilitation training (saliva swallowing after treatment minus that at acute period, Figure 6).

## 4 Discussion

In this study, we investigated cortical activation during saliva swallowing and effortful saliva swallowing using BOLD imaging shortly after MI and after dysphagia recovery. All patients in our study had dorsolateral medulla damage and relatively severe swallowing difficulties, and some had indwelling gastric tubes. In these patients, swallowing water may increase the risk of choking; therefore, saliva swallowing was chosen as the BOLD paradigm. Some studies have used single-swallow saliva designs for their experiments (4); however, the period was too short for some patients to complete a swallow. Thus, the present study examined continuous saliva swallowing over a longer period. In addition, some experiments evaluated continuous saliva swallowing for 30 s (9). However, in our preliminary tests, we observed that after several consecutive swallows, there was little saliva left to swallow, and additional actions may be required to produce more saliva, which may cause a change in the activated areas. Therefore, an 18 s design was selected, allowing participants to have enough time to rest and produce more saliva.

### 4.1 Activated regions during saliva swallowing in the acute phase of stroke and insula activation

In this study, very few brain regions were activated during saliva swallowing in the acute phase of stroke. Specifically, only the bilateral precentral gyri and left lingual gyrus were partially activated. This is similar to the findings of previous studies (9), and the right inferior semilunar lobule and right posterior lobe of the cerebellum were slightly activated. The precentral gyrus is the activated cortex for voluntary movement, whereas the lingual and inferior occipital gyri form a visual association cortex that may have been associated with the visual swallowing cues in this study. The inferior semilunar lobule, a new lobe of the cerebellum located in the posterior lobe of the cerebellum, receives cortical pontine fibers and exhibits very little activation, which may be related to the coordinated movement of swallowing actions. Although the patients only had a bulbar injury, the small number of activated brain areas indicates that the bulbar region is an important component of the swallowing network (10, 11) and that it influences the related brain regions of the swallowing network. Notably, there was a lack of general cortical activation during swallowing.

Currently, only a few studies have examined brain activation patterns in patients with post-stroke dysphagia. Some studies have shown a significant decrease in the cortical activation areas (9, 12, 13); however, Li et al. reported increased activation in swallowing-related regions, including the left precentral gyrus, left postcentral gyrus, left SMA, and insula (14). Further, Zhou et al. observed a significant reduction in the activation of the anterior cingulate gyrus and excessive activation in other non-swallowing-related regions, including the posterior cingulate gyrus, parahippocampal cortex, visual center, and primary auditory center (15). These inconsistent results may be due to differences between the selected participants. For example, mild-to-moderate dysphagia caused by unilateral hemisphere stroke can produce compensation in the unaffected side or other brain regions (14, 15). However, in the present study, all the patients experienced medullary damage, which is a crucial component of the swallowing network. The medulla participants pharyngeal swallowing and requires bilateral coordination, making functional compensation challenging. Consequently, most cerebral regions cannot be activated, and severe dysphagia is clinically observed. In this study, the patients were in the acute phase of stroke, and cortical activation was scarce, similar to patients with brainstem damage 1–12 months after stroke (9). It is speculated that the activation of the cortex during swallowing in patients with dysphagia is more related to the damaged region and degree than to the post-injury period. If the brainstem damage does not recover, most cortices will exhibit decreased activation regardless of the time after injury.

To date, the conclusions of various studies regarding insular activation in patients with dysphagia have been inconsistent. Patients with unilateral cortical and subcortical infarctions and mild dysphagia exhibited bilateral insular activation (14, 15) with increased activation of the undamaged insula compared with patients in the control group (14), whereas other studies reported no insular activation (9, 12, 13). In this study, all patients had pharyngeal dysphagia, and no insular activation was observed in the acute phase; however, bilateral insular activation was observed after recovery. This finding suggests that the impairment of insular activation may be related to pharyngeal dysphagia. The insula contributes to the initiation of swallowing by processing sensory elements and interacting with widespread brain regions in both hemispheres, including the primary sensorimotor cortex (16). The frontal insula is believed to regulate the temporal organization of mouth movements, such as the timing of the onset of swallowing after a series of chewing or other mouth movements (5). The absence of insular activation may affect the patient's ability to plan swallowing movements, leading to uncoordinated or abnormal swallowing completion, which is manifested as a delay in the start of pharyngeal swallowing. Activation of the insula maybe a compensatory mechanism for the recovery of swallowing function.

### 4.2 Increased activation of brain regions during swallowing after treatment

Only a few studies have compared the activated brain areas during swallowing before and after rehabilitation. Wei et al. found that the volume of activation in the affected hemisphere increased significantly after rehabilitation, whereas there was no change or only a slight increase in the unaffected hemisphere. Therefore, the recovery of cerebral hemisphere function on the affected side is important for the rehabilitation of patients with swallowing disorders (13). In contrast, Li et al. observed an increase in activation in the contralateral hemisphere during the acute phase of stroke and suggested that compensation from the intact side promoted functional recovery (14). These inconsistent results may be attributed to the heterogeneity of the enrolled patients. To increase accuracy, it is advisable to select relatively consistent patients, such as those with unilateral involvement of a certain cortical or subcortical structure.

After language training, patients with dysphagia caused by unilateral hemisphere stroke exhibited significant activation of the bilateral primary motor areas, sensory areas, premotor areas, and the anterior insula during swallowing (6). In patients with brainstem damage, increased activation of brain regions, including the cingulate gyrus, insula, precuneus, frontal lobe, orbitofrontal cortex, and SMAs, was observed after treatment. These findings suggest that other brain regions have some compensatory ability when a swallowing center injury occurs (9). Our study demonstrated the bilateral precentral gyrus, postcentral gyrus, insula, thalamus, and SMA showed increased activation after rehabilitation training. These regions are not completely consistent with the above results (9), which may be attributed to different post-injury periods and different compensatory mechanisms between the early and chronic periods.

Mihai et al. found that even after swallowing function recovery, the overall activation level of swallowing-related brain regions in patients was still lower than that of the control group (17). Meanwhile, there was increased activation in the contralateral S1 area; the more obvious the initial damage, the more obvious the activation in the contralateral S1 area after functional recovery. This indicates that activation of the contralateral S1 area is the reason for the recovery of swallowing function. Domin et al. revealed that the integrity of callosal fibers interconnecting the S1 swallowing representation were significantly associated with swallowing compliance (18). An increase in activation was observed in the postcentral gyrus in this study, which is similar to previous results (17, 18). This suggests that after the motor function is impaired, increased somatosensory input is required to complete swallowing tasks; that is, sensory reorganization contributes to compensation.

Oral sensory stimulation can activate the sensory and motor areas associated with swallowing, which can enhance sensory input and participate in the control center of the brainstem and cortex, potentially affecting the autonomous components of swallowing (19). Studies have revealed that pharyngeal stimulation first activates the sensory-motor cortex, then excites the swallowing motor cortex and changes the recruitment pattern of cortical activation related to swallowing (20, 21). In this study, all patients had pharyngeal dysphagia, and FEES revealed decreased sensation in the pharynx. When the probe touched the epiglottis and posterior pharyngeal wall, it did not elicit a swallowing reflex, causing a delay in the swallowing response; this is an important cause of choking. The patients in this study underwent ice-cotton swab stimulation training, which improved pharyngeal sensation and increased sensory input to the postcentral gyrus, possibly by activating the thalamus. Sensory compensation contributed to clinical recovery, as confirmed using FEES, which showed improved pharyngeal sensation after recovery. This finding suggests that the tactile input during swallowing may be a crucial factor in recovery. In the treatment of dysphagia, especially pharyngeal dysphagia, it is necessary to focus not only on motor training but also on sensory stimulation training to better promote swallowing function.

### 4.3 Comparison of brain activation between effortful saliva swallowing and saliva swallowing before and after treatment

After treatment, there was only a slight increase in activation in the left SMA, right supramarginal gyrus, and left postcentral gyrus when effortful saliva swallowing was compared with saliva swallowing. Peck et al. found that healthy individuals exhibited increased activation in the angular gyrus, cingulate gyrus, inferior parietal lobule, middle frontal gyrus, superior frontal gyrus, and supramarginal gyrus when comparing effortful saliva swallowing to saliva swallowing (22). The results of the present study were similar, although the range of differences in activation was smaller. However, before treatment, a significant increase was observed in brain activation between the two swallowing actions, including the bilateral SMA, bilateral postcentral gyri, and right insula. This finding suggests that patients with acute medullary stroke need to exert more effort to complete effortful saliva swallowing and activate more brain regions, which are the compensatory areas activated when normal saliva swallowing is performed after recovery.

Gandhi et al. (23) found that effortful swallowing can improve swallowing function in patients with PD more effectively than normal swallowing, whereas Kim et al. (24) found that tongue-to-palate training can increase the oral-pharyngeal swallowing function of subacute stroke patients. The results of these two studies are consistent with our findings. Additionally, Bahia et al. reviewed 23 studies and found that the biomechanical effects of effortful swallowing include increased pressure in the oral, pharyngeal, and esophageal regions (25). The BOLD imaging results confirmed that the recovery of swallowing function after stroke can be attributed to the compensation of more brain regions; however, the results cannot confirm that rehabilitation training itself stimulates the compensatory brain regions. Here, we observed that effortful swallowing after MI can activate these compensatory brain regions and promote faster recovery of swallowing function. This indicates that in pharyngeal dysphagia caused by MI, effortful saliva swallowing can activate more brain regions and better promote the recovery of swallowing function, making it a more suitable rehabilitation training method than saliva swallowing.

## 5 Study limitations and future directions

This study has some limitations. First, to ensure the comfort of patients during BOLD study, saliva swallowing and effortful saliva swallowing actions were observed subjectively by the experimenters without objective quality control. Nevertheless, documenting the quality control of the performance of participants during fMRI is very important (2). In future studies, electromyography or pads placed on the neck to record the thyroid cartilage motion could be used to mark swallowing. Second, FEES was only used to confirm that the enrolled patients had pharyngeal dysphagia at the initial design of the experiment, so no numerical scoring was performed. However, if FEES scoring was available for the improvement of pharyngeal sensation, it would be more accurate. Third, only 15 patients were enrolled, and the MI was not differentiated between the left and right sides, making it impossible to distinguish whether the contralesional or ipsilesional hemispheres were more compensatory. In the future, increasing the number of trial participants and grouping them according to the injured side can ensure more accurate observation of compensatory mechanisms.

Although the Mendelson maneuver can also be used to increase cortical activation in healthy individuals (22), it requires a 3 s breath hold after lifting the larynx; this could not be accurately controlled in this study. Moreover, breath-holding during MRI may pose a risk for patients with MI. In the future, such training could be examined under the premise of ensuring patient safety. Jing et al. found that observing swallowing movements could activate mirror neurons and swallowing networks in healthy individuals, indicating the potential value of action observation in the treatment of swallowing disorders (26). We can observe whether this safe training technique is useful for patients with dysphagia in the future.

## 6 Conclusion

This study demonstrated that patients with dysphagia after MI did not exhibit insular activation at the acute stroke period. After recovery, activation of the posterior central gyrus increased, which is consistent with the improved swallowing function and pharyngeal sensation in FEES. The BOLD results confirmed that the sensory cortex might play a compensatory role in the recovery of swallowing function. Simultaneously, effortful saliva swallowing after MI significantly increased activation in swallowing-related regions, and these brain regions promoted the recovery of swallowing function, suggesting that effortful saliva swallowing is a more effective rehabilitation training method for patients with dysphagia after MI.

## Data availability statement

The data analyzed in this study is subject to the following licenses/restrictions: The raw data is the result of fMRI, which is saved in the form of a CD and requires software analysis. The figures and tables attached in the article can be used publicly, but only members of the research team can view the original MRI images. Requests to access these datasets should be directed to FG, googledragon@163.com.

## Ethics statement

The studies involving humans were approved by Ethics Committee of Tianjin Huanhu Hospital. The studies were conducted in accordance with the local legislation and institutional requirements. The participants provided their written informed consent to participate in this study.

## Author contributions

FG: Data curation, Investigation, Methodology, Writing – original draft, Conceptualization, Writing – review & editing. JH: Data curation, Investigation, Writing – original draft. QZ: Data curation, Investigation, Writing – original draft. XL: Methodology, Writing – original draft. YW: Data curation, Investigation, Writing – original draft. JW: Project administration, Supervision, Writing – review & editing, Methodology.
